# Undertaking a research project improves confidence in research skills among student dietitians

**DOI:** 10.1111/jhn.13004

**Published:** 2022-04-07

**Authors:** Kevin Whelan, Kate R. Castelli, Camilla Trizio, Oliver Howard, Jane E. Thomas, Angela M. Madden

**Affiliations:** ^1^ Department of Nutritional Sciences King's College London London UK; ^2^ School of Life & Medical Sciences University of Hertfordshire Hatfield UK

**Keywords:** dietetics, dietitian, education, research, student

## Abstract

**Background:**

Research is a cornerstone of evidence‐based dietetic practice. Research skills are often taught at university through experiential learning during a final‐year research project. The present study aimed to investigate attitudes towards research and confidence in research skills among student dietitians before and after a research project.

**Methods:**

A questionnaire survey of student dietitians’ attitudes to research and confidence in research skills was undertaken before and after completing a research project at two universities in London, UK. Dichotomous data were compared before and after the research project using a McNemar's test. Factors associated with ‘high confidence’ or ‘improved confidence’ in overall research skills at the end of the research project were investigated using multivariable logistic regression.

**Results:**

In total, 160 student dietitians completed a questionnaire before and after their research project. The majority had positive attitudes to research both before and after their research project. There was an increase in numbers with ‘high confidence’ in overall research skills before (13; 8.1%) and after (79; 49.4%) the research project (*p* < 0.001), and 113 (70.6%) reported ‘improved confidence’ in overall research skills. The only factor associated with ‘high confidence’ in overall research skills was having ‘high levels of involvement in the overall research process’ (odds ratio = 6.13, 95% confidence interval = 2.03–18.49, *p* = 0.001).

**Conclusions:**

Student dietitians have positive attitudes towards research and undertaking a research project significantly improves confidence in their research skills. A higher level of involvement in the research project is the single most significant factor associated with high confidence in research skills.

## Key points

- This is the largest study to date of student dietitians’ experiences of undertaking a research project during their university degree.
- Student dietitians have positive attitudes to research both before and after their research project.
- Research projects improve student dietitians’ confidence in their research skills.
- The single most significant factor associated with developing high confidence in research skills is having a greater level of involvement in the project.

## Graphical abstract

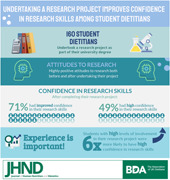

## INTRODUCTION

Research is a cornerstone of evidence‐based dietetic practice. Research is essential to provide the evidence for diet–disease relationships, for the effectiveness of interventions and to provide evidence of the impact of dietitians.^1^ Research builds a knowledge base for the relatively new discipline of nutrition. It is crucial for the advancement of the dietetic profession^2^, ^3^ and for the credibility and public perception of dietitians.^4^ Meanwhile, healthcare professionals’ involvement in research in the clinical setting can benefit clinical practice, including improvements in health services performance, processes of care and patient outcomes, as a result of having greater knowledge of research studies, implementation of guidelines, and greater networks and collaborative working.^5^

Professional associations or governing bodies have standards for dietitians that include competency in research and evidence‐based practice in Australia,^6^ Canada,^7^ UK,^8^ USA^9^ and many other countries across the world, and are also included in the International Practitioner Competency Standards for Dietitians set by the International Confederation of Dietetic Associations.^10^

In the wider healthcare professions, generally positive attitudes towards involvement in research have been reported.^11^, ^12^ This is also true in small studies of dietitians^13^, ^14^ and student dietitians.^15^, ^16^ However, there remains a significant gap in healthcare professionals’ attitudes to research and their actual participation in research.^17^, ^18^ Studies report variable levels of research involvement among dietitians in Australia,^19^ Canada,^20^ UK^14^ and USA.^1^, ^21^, ^22^, ^23^

Barriers to research involvement in dietetics have been extensively examined and consistently report a lack of time and lack of funding as key issues, but negative attitudes towards research and lack of confidence in research skills have also been identified as important obstacles to research involvement.^21^, ^22^, ^24^ In particular, limited pre‐registration training in research was reported as a barrier to dietitians subsequently undertaking research in a survey of the Academy of Nutrition and Dietetics,^22^ and is consistent with other studies in the USA^1^, ^21^, ^25^ and UK.^14^

The reported lack of sufficient research training in pre‐registration dietetics programmes coupled with evidence that education, knowledge and interest in research are associated with future research involvement among dietitians^23^, ^24^ is concerning because today's students will be tomorrow's profession who will be responsible for undertaking, applying and disseminating future research.^26^

In the UK, pre‐registration programmes in dietetics require student dietitians to have a ‘critical and applied knowledge and understanding of research …’ including, amongst others: research ethics and research governance; the principles of scientific enquiry; quantitative and qualitative research design; data management; statistical analysis and interpretation; critical appraisal; and evidence informed practice.^27^ At most universities in the UK, research skills training culminates in the student undertaking a research project usually in the final year of the programme. Exposing students to the research process is supported by an educational theory of experiential learning, a model in which learners gain skills and knowledge from direct experience.^28^, ^29^

A small number of studies have demonstrated that student dietitians’ exposure to undertaking a research project improves confidence in their research skills.^3^, ^15^, ^30^ However, little is known of the change in attitudes and confidence resulting from undertaking a research project, nor the factors associated with optimal outcome, mainly as a result of the lack of before and after analyses and limited cohort sizes.

The present study aimed to investigate attitudes towards research and confidence in research skills among student dietitians before and after a research project. Specifically, the study investigates: (i) attitudes towards research and confidence in research skills among student dietitians; (ii) the effect of undertaking a final year research project on these attitudes and confidence; and (iii) the factors associated with ‘high confidence’ and ‘improved confidence’ in overall research skills.

## METHODS

The study used a questionnaire to investigate the confidence and attitudes of student dietitians before and after undertaking their final‐year research project.

### Participants

Data were collected from students before and after completing a compulsory research project module in their final year of the 4‐year BSc Nutrition and Dietetics programmes at King's College London and London Metropolitan University, two large universities in London, UK. All students undertaking the research project module were eligible, with no exclusion criteria, and all were approached over five consecutive years at King's College London (academic years ending in 2005–2009) and three consecutive years at London Metropolitan University (academic years ending in 2005–2007), aiming to recruit a sufficiently large, unselected and representative sample.

### Intervention

Both universities provide students with a compulsory ‘research methods’ module earlier in the BSc degree (Years 1 or 2) in preparation for the research project module in the final (Year 4). The research project modules involve some research methods teaching (e.g., study design, research ethics, data analysis) followed by undertaking a research project part‐time and under supervision for 3–4 months.

Project selection consisted of compilation of a list of research projects proposed by academic staff at the university or by an external dietitian or nutritionist who would co‐supervise a project with an internal academic.^31^ Students were provided with the list of proposed projects that included the email address of the supervisor(s) who could be contacted for further information regarding the project if required. Projects were all relevant to nutrition and dietetics and were diverse in the type of research activity (e.g., literature review or systematic review, experimental laboratory, questionnaire survey or dietary survey, clinical audits, clinical trial or feeding study). Some projects involved original data collection, whereas others involved analysing data that had already been collected. Students rated their preferred research project(s) and allocation was based not only primarily upon their expressed preference, but also with a goal to distribute supervisory load between academic staff.

Following project allocation, all research projects were conducted according to standard university procedures. A research team was formed consisting of the student dietitian and one (or more) university academic and, for external collaborative projects, the external dietitian/nutritionist. Each research team had a preliminary meeting to discuss the project, team member responsibilities, supervisory arrangements and the dissemination of results.

On completion of the research projects, students wrote a dissertation and prepared and presented a research presentation or poster or participated in a viva voce in line with the assessment requirements of their university.

### Questionnaire

Students were invited to participate in a survey of their attitudes and confidence in their research skills by completing a questionnaire before (first lecture of the module) and after undertaking their final‐year research project (following submission of their dissertation and completion of their assessment). The questionnaire was developed by the three members of the research team (KW, JET and AMM) with extensive experience of questionnaire design, psychometric testing and survey conduct. Questionnaire design consisted of performing a literature search to identify previously conducted surveys, where possible, based upon which a bespoke questionnaire was developed. The first iteration of the questionnaire was piloted in 36 students and amendments were made following feedback and discussion, and the data collected from the pilot questionnaire were not included in the current analysis.

Attitudes towards research were measured using a series of nine statements reflecting a range of positive and negative attitudes towards research and were taken directly from a previous questionnaire, which, at the time of the study design, was one of the few studies of dietitians’ attitudes towards research.^14^ The only amendment was to two statements: ‘Clinical research should be led by clinicians’, which was clarified to ‘Clinical research should be led by clinicians (doctors)’ to ensure consistency of interpretation, and ‘I don't see research as part of my job’, which was changed to ‘I don't see research as part of a dietitian's job’ to reflect the respondents’ status as students. Each statement was rated using a five‐point Likert scale (‘1, Strongly agree’, ‘5, Strongly disagree’), as in the previous questionnaire.^14^ As well as presenting data on the level of agreement with each statement, data were collapsed to show the numbers with positive attitudes depending on the framing of statements. For example, rating ‘Agree’ or ‘Strongly Agree’ to a positive statement (e.g., ‘Research should be carried out by all dietitians’) or ‘Disagree’ or ‘Strongly Disagree’ to a negative statement (e.g., ‘I don't see research as part of a dietitian's job’) were both considered to reflect positive attitudes. Data were also used to calculate the numbers whose attitudes improved between baseline and after completing the project (e.g., whose answers moved in a ‘more positive’ direction). Attitudes towards future involvement in research were measured on a dichotomous Yes/No scale.

Confidence in research skills was measured for 10 research activities based upon the different stages of research process from a model provided by the National Institute of Health Research at the time (e.g., ‘Developing a hypothesis’, ‘Designing a research protocol’, and ‘Drawing conclusions from research’). Minor adaptations were made; namely, removing a question on obtaining research funding (which is not usually required as part of university research projects): changing ‘Report on the study and disseminate the findings’ into two processes ‘Writing a research report’ and ‘Orally presenting research findings’ (as both were performed as part of the research project modules and require distinct sets of skills); and, finally, a question regarding confidence in the ‘Overall research process’ was added (to encompass overall confidence in research and not just in a specific stage of the research process). Confidence was rated on a five‐point Likert scale (‘1, Not at all confident’, ‘5, A great deal of confidence’). As well as presenting data on confidence in each of the 10 activities and in the overall research process, data were also collapsed to report those with ‘high confidence’ (‘4, A lot of confidence’, ‘5, A great deal of confidence’). The numbers of students with ‘improved confidence’ were calculated, defined as increasing in confidence by one or more on the Likert scale between baseline and after completing the project (e.g., changing from ‘2, A little confidence’ to ‘3, Quite a bit of confidence’).

The questionnaire completed at the end of the research project also examined the extent to which students were involved in different aspects of research during their project and their perceptions of whether the project had contributed to the development of their research skills. These two outcomes were measured for the 10 research activities and the overall research process and were rated using the same five‐point Likert scale (‘1, Not at all’, ‘5, A great deal’). High levels of involvement were defined as the student reporting ‘4, A lot of involvement’ or ‘5, A great deal of involvement’ in the research project.

### Ethical considerations

The study received ethical approval from the research ethics committees of both King's College London and London Metropolitan University. Questionnaires were distributed during lectures in sealed envelopes and completed on a voluntary basis. Questionnaires were anonymised using a code such that questionnaires before and after the research project could be paired for analysis but did not contain the students’ name or any identifiable details. Questionnaire responses were not linked in any way to student records or academic results and did not contribute or impact in anyway on the assessment of the research project module.

### Statistical analysis

Data were only analysed for students who provided a questionnaire at both baseline and following completion of the project, in order that changes in attitudes and confidence could be calculated for all respondents. Descriptive statistics were calculated for the variables of interest and data are presented as *n* (%). Dichotomised data were compared between baseline and after the research project using a McNemar's test.

The two major educational outcomes of interest were having ‘high confidence’ in overall research skills after the research project (coded as either ‘4, A lot of confidence’, ‘5, A great deal of confidence’ on the questionnaire at the end of the project) and ‘improved confidence’ in overall research skills between baseline and after the research project (coded as an increase in confidence of one or more on the Likert scale between baseline and end of the research project). These two educational outcomes were explored for related factors using univariable logistic regression. Eight variables were entered into the model relating to personal characteristics (age, gender, university, previous involvement in research) and project‐related characteristics (supervision model/location, project type, original data collection, student's reported involvement in the overall research process after the project). Personal factors with a *p* < 0.1 in the univariate logistic regression model together with all project‐related characteristics were included in the multivariable logistic regression model.

Data were analysed in SPSS, version 26 (IBM Corp.). *p* < 0.05 was considered statistically significant.

## RESULTS

### Participant characteristics

Questionnaires were distributed to 196 students, of whom 184 (93.9%) returned a questionnaire at baseline and 166 (84.7%) returned one following completion of the research project. In total, 160 (81.6%) students returned a questionnaire at both time points and are the complete case analysis study population reported here.

The 160 students had a median age of 25.0 years (interquartile range = 10.7) and 149 (93.1%) were female (Table 1). Overall, 115 (71.9%) studied at King's College London and 45 (28.1%) studied at London Metropolitan University. Two‐thirds (66.3%) undertook an internal project (based at the university and supervised by a university academic) and one‐third (33.7%) undertook an external project (based in a hospital, clinic or other research institute, and supervised by both a university academic and an external dietitian/nutritionist). A wide range of project types were undertaken, including questionnaires or dietary surveys (30.0%), literature reviews or systematic reviews (23.8%) and experimental laboratory projects (18.1%) (Table 1).

**Table 1:** Characteristics of the 160 student dietitians participating in the final year research project study

**Characteristics**	**Data**
Age, years, median (interquartile range)	25.0 (10.7)
Gender, *n* (%)	
Female	149 (93.1)
Male	11 (6.9)
University, *n* (%)	
King's College London	115 (71.9)
London Metropolitan University	45 (28.1)
Previous research involvement, *n* (%)	
No	134 (83.8)
Yes	26 (16.2)
Supervision model, *n* (%)	
Internal project and supervisor	106 (66.3)
External project with internal and external supervisor	54 (33.7)
Project type, *n* (%)	
Literature review or systematic review	38 (23.8)
Experimental laboratory	29 (18.1)
Questionnaire or dietary survey	48 (30.0)
Clinical audit	24 (15.0)
Clinical trial or feeding study	21 (13.1)

### Attitudes toward research

The majority of student dietitians’ responses to statements reflected positive attitudes towards research at baseline and after completing the project (Table 2). The only exception was ‘seeing patients is more important than research’. There were no significant differences in the numbers with positive attitudes between baseline and after the research project, except for ‘I find it hard to interpret research’, for which there was an increase in number of students who ‘disagreed’ or ‘strongly disagreed’ between baseline (44.4%) and after the research project (61.8%, *p* < 0.001). The numbers whose attitudes improved after the research project ranged from 10.6% (‘I find it hard to interpret research’) to 26.3% (‘I don't see research as part of a dietitian's job’). In terms of attitudes towards future involvement in research, after the research project, a very small minority of students (5.6%) said they ‘did not want to be involved in audit and research’ and the majority (63.1%) said they wanted ‘to take part in research with colleagues’ (Table 3).

**Table 2:** Comparison of attitudes towards research before and after undertaking a research project among 160 student dietitians

		**Level of agreement with statements**, ***n*** **(%)**					**Positive attitude** a		**Improved attitude** b
**Attitude statement**		Strongly agree	Agree	Neither agree nor disagree	Disagree	Strongly disagree	*n* (%)	*p* value	*n* (%)
Research should be carried out by all dietitians	Baseline	19 (11.9)	71 (44.4)	41 (25.6)	28 (17.5)	1 (0.6)	90 (56.3)	0.542	34 (21.3)
	After research project	28 (17.5)	67 (41.9)	39 (24.4)	22 (13.8)	4 (2.5)	95 (59.4)		
Research should only be carried out by research dietitians	Baseline	8 (5.0)	16 (10.0)	25 (15.6)	85 (53.1)	26 (16.3)	111 (69.4)	0.760	40 (25.0)
	After research project	0 (0.0)	12 (7.5)	40 (25.0)	79 (49.4)	29 (18.1)	108 (67.5)		
All dietitians should be able to act on research	Baseline	72 (45.0)	75 (46.9)	11 (6.9)	2 (1.3)	0 (0.0)	147 (91.9)	0.424	22 (13.8)
	After research project	81 (50.6)	70 (43.8)	6 (3.8)	2 (1.3)	1 (0.6)	151 (94.4)		
Doing research improves patient care	Baseline	70 (43.8)	71 (44.4)	16 (10.0)	3 (1.9)	0 (0.0)	141 (88.1)	0.327	30 (18.8)
	After research project	67 (41.9)	80 (50.0)	11 (6.9)	1 (0.6)	1 (0.6)	147 (91.9)		
I don't see research as part of a dietitian's job	Baseline	2 (1.3)	7 (4.4)	30 (18.8)	69 (43.1)	52 (32.5)	121 (75.6)	0.651	42 (26.3)
	After research project	2 (1.3)	7 (4.4)	26 (16.3)	78 (48.8)	47 (29.4)	125 (78.1)		
Seeing patients is more important than research	Baseline	3 (1.9)	27 (16.9)	72 (45.0)	49 (30.6)	9 (5.6)	58 (36.3)	0.263	38 (23.8)
	After research project	5 (3.1)	17 (10.6)	71 (44.4)	58 (36.3)	9 (5.6)	67 (41.9)		
Research provides evidence to direct patient care	Baseline	78 (48.8)	66 (41.3)	12 (7.5)	1 (0.6)	3 (1.9)	144 (90.0)	0.115	32 (20.0)
	After research project	70 (43.8)	82 (51.3)	5 (3.1)	2 (1.3)	1 (0.6)	152 (95.0)		
Clinical research should be led by clinicians (doctors)	Baseline	1 (0.6)	6 (3.8)	38 (23.8)	80 (50.0)	35 (21.9)	115 (71.9)	0.575	37 (23.1)
	After research project	0 (0.0)	4 (2.5)	36 (22.5)	83 (51.9)	37 (23.1)	120 (75.0)		
I find it hard to interpret research findings	Baseline	7 (4.3)	38 (23.8)	44 (27.5)	61 (38.1)	10 (6.3)	71 (44.4)	< 0.001	17 (10.6)
	After research project	2 (1.3)	15 (9.4)	44 (27.5)	83 (51.9)	16 (10.0)	99 (61.8)		

^a^ Numbers (%) with positive attitudes depending on the framing of statements. For example, positive attitudes were considered to be rating ‘Agree’ or ‘Strongly Agree’ to a positive statement (e.g., ‘Research should be carried out by all dietitians’) or ‘Disagree’ or ‘Strongly Disagree’ to a negative statement (e.g., ‘I don't see research as part of a dietitian's job’). The *p* value represents comparison of the numbers with positive attitudes at baseline and after research project using McNemar's test

^b^ Number (%) whose response improved in favour of a more positive attitude towards research after completing a research project.

**Table 3:** Comparison of attitudes towards future involvement in research before and after undertaking a research project among 160 student dietitians

**Attitude statement**	**Baseline**, ***n*** **(%)**	**After research project**, ***n*** **(%)**	*p* **value**
I do not want to be involved in audit or research	14 (8.8)	9 (5.6)	0.359
I expect I would have to be involved in audit activities	65 (40.6)	64 (40.0)	1.000
I want to be involved in developing audit projects	40 (25.0)	52 (32.5)	0.127
I want to take part in research with colleagues	80 (50.0)	101 (63.1)	0.009
I hope to be involved in audit and research projects	57 (35.6)	78 (48.8)	0.01
I want to be more involved in research projects	51 (31.9)	61 (38.1)	0.175
I would consider pursuing a higher degree	55 (34.4)	52 (32.5)	0.735

*p* value represents comparison of the responses at baseline and after research project using McNemar's test

After completing the research project, there were no differences for five of the seven attitude statements regarding future involvement in research. However, there was an increase in the numbers who ‘hope to do audit and research’, (baseline 35.6% vs. after project 48.8%, *p* = 0.01) and ‘hope to do research with colleagues’ (baseline 50% vs. after project 63.1%, *p* = 0.009) (Table 3).

### Confidence in research skills

The number of students with high confidence (‘4, A lot of confidence’, ‘5, A great deal of confidence’) in their research skills was very low at baseline, ranging from only 6.9% (‘statistical analysis’) to 35.6% (‘orally presenting research findings’) (Table 4). However, after completing the research project, there were significantly greater numbers with high confidence for nine of the 10 research skills. This resulted in the majority of students having high confidence in five of the 10 research skills: ‘completing a literature search’ (82.5%), ‘collecting new data’ (52.5%), ‘writing a research report’ (77.5%), ‘drawing conclusions from research’ (76.3%) and ‘orally presenting research findings’ (65.6%).

**Table 4:** Confidence in research skills before and after undertaking a research project among 160 student dietitians

		**Level of confidence in research skills**, ***n*** **(%)**					**High confidence** a		**Improved confidence** b
**Research activity**		Not at all	A little	Quite a bit	A lot	A great deal	*n* (%)	*p* value	
Developing a hypothesis	Baseline	27 (16.8)	64 (40.0)	47 (29.4)	17 (10.6)	5 (3.1)	22 (13.8)	< 0.001	89 (55.6)
	After research project	10 (6.3)	30 (18.8)	58 (36.3)	45 (28.1)	17 (10.6)	62 (38.8)		
Completing a literature search	Baseline	5 (3.1)	37 (23.1)	62 (38.8)	44 (27.5)	12 (7.5)	56 (35.0)	< 0.001	114 (71.3)
	After research project	1 (0.6)	3 (1.9)	24 (15.0)	72 (45.0)	60 (37.5)	132 (82.5)		
Deciding on data collection methods	Baseline	31 (19.4)	68 (42.5)	44 (27.5)	13 (8.1)	4 (2.5)	17 (10.6)	< 0.001	95 (59.4)
	After research project	10 (6.3)	38 (23.8)	56 (35.0)	39 (24.4)	17 (10.6)	56 (35.0)		
Designing a research protocol	Baseline	31 (19.4)	62 (38.8)	48 (30.0)	15 (9.4)	4 (2.5)	19 (11.9)	< 0.001	90 (56.3)
	After research project	13 (8.1)	39 (24.4)	52 (32.5)	44 (27.5)	12 (7.5)	56 (35.0)		
Obtaining research ethics approval	Baseline	80 (50.0)	52 (32.5)	15 (9.4)	9 (5.6)	4 (2.5)	13 (8.1)	0.124	46 (28.8)
	After research project	81 (50.6)	41 (25.6)	16 (10.0)	15 (9.4)	7 (4.4)	22 (13.8)		
Collecting original data	Baseline	24 (15.0)	64 (40.0)	56 (35.0)	13 (8.1)	3 (1.9)	16 (10.0)	< 0.001	100 (62.5)
	After research project	21 (13.1)	24 (15.0)	31 (19.4)	55 (34.4)	29 (18.1)	84 (52.5)		
Statistical analysis	Baseline	47 (29.4)	63 (39.4)	39 (24.4)	8 (5.0)	3 (1.9)	11 (6.9)	< 0.001	85 (53.1)
	After research project	32 (20.0)	31 (19.4)	51 (31.9)	31 (19.4)	15 (9.4)	46 (28.8)		
Drawing conclusions from research	Baseline	12 (7.5)	44 (27.5)	69 (43.1)	29 (18.1)	6 (3.8)	35 (21.9)	< 0.001	120 (75.0)
	After research project	4 (2.5)	2 (1.3)	32 (20.0)	78 (48.8)	44 (27.5)	122 (76.3)		
Writing a research report	Baseline	9 (5.6)	50 (31.3)	73 (45.6)	25 (15.6)	3 (1.9)	28 (17.5)	< 0.001	121 (75.6)
	After research project	2 (1.3)	5 (3.1)	29 (18.1)	68 (42.5)	56 (35.0)	124 (77.5)		
Orally presenting research findings	Baseline	6 (3.8)	36 (22.5)	61 (38.1)	46 (28.8)	11 (6.9)	57 (35.6)	< 0.001	92 (57.5)
	After research project	6 (3.8)	10 (6.3)	39 (24.4)	55 (34.4)	50 (31.3)	105 (65.6)		
Overall research process	Baseline	15 (9.4)	60 (37.5)	72 (45.0)	10 (6.3)	3 (1.9)	13 (8.1)	< 0.001	113 (70.6)
	After research project	3 (1.9)	9 (5.6)	69 (43.1)	59 (36.9)	20 (12.5)	79 (49.4)		

^a^ Numbers (%) with ‘High confidence’ are those reporting either ‘A lot’ or ‘A great deal’ of confidence in their research skills. *p* value represents comparison of the numbers with high confidence at baseline and after research project using McNemar's test

^b^ Number (%) with ‘improved confidence’ defined as increasing in confidence by one or more on the Likert scale between baseline and after completing the project (e.g., changing from ‘2, A little confidence’ to ‘3, Quite a bit of confidence’).

There was also a significant increase in the numbers with high confidence in the ‘overall research process’ increasing from 8.1% at baseline to 49.4% after the research project. More than half of the students showed improved confidence in their research skills for nine of the 10 research skills (ranging from 53.1% ‘statistical analysis’ to 75.6% ‘writing a research report’). The exception was ‘obtaining research ethics approval’ (28.8%).

### Involvement in research project and perceptions of role in skill acquisition

The areas of the research project in which the most students had high levels of involvement (‘4, A lot of involvement’, ‘5, A great deal of involvement’) were ‘completing a literature search’ (91.3%), ‘writing a research report’ (95.6%) and ‘drawing conclusions from research’ (95.0%). The areas where fewest students had high levels of involvement were ‘obtaining research ethics approval’ (11.3%), ‘designing a research protocol’ (42.5%) and ‘deciding on data collection methods’ (38.8%). In total, 133 (83.1%) students reported high levels of involvement in the overall research process (Table 5).

**Table 5:** Level of involvement in aspects of the research project and perception of the extent to which the research project contributed to the development of their research skills among student dietitians

	**Level of involvement in this aspect of the research project**, ***n*** **(%)**					**Perception of the extent to which undertaking the research project contributed to the development of their research skills**, ***n*** **(%)**				
**Research activity** a	Not at all	A little	Quite a bit	A lot	A great deal	Not at all	A little	Quite a bit	A lot	A great deal
Developing a hypothesis	33 (20.6)	28 (17.5)	28 (17.5)	26 (16.3)	45 (28.1)	15 (9.4)	35 (21.9)	39 (24.4)	48 (30.0)	23^14^
Completing a literature search	1 (0.6)	1 (0.6)	14 (8.8)	31 (19.4)	113 (70.6)	2 (1.3)	8 (5.0)	27 (16.9)	56 (35.0)	67 (41.9)
Deciding on data collection methods	47 (29.4)	28 (17.5)	23 (14.4)	22 (13.8)	40 (25.0)	19 (11.9)	32 (20.0)	44 (27.5)	45 (28.1)	20 (12.5)
Designing a research protocol	41 (25.6)	33 (20.6)	18 (11.3)	27 (16.9)	41 (25.6)	20 (12.5)	33 (20.6)	40 (25.0)	42 (26.3)	25 (15.6)
Obtaining research ethics approval	125 (78.1)	8 (5.0)	9 (5.6)	8 (5.0)	10 (6.3)	100 (62.5)	29 (18.1)	13 (8.1)	8 (5.0)	10 (6.3)
Collecting original data	43 (26.9)	9 (5.6)	18 (11.3)	15 (9.4)	75 (46.9)	31 (19.4)	19 (11.9)	21 (13.1)	51 (31.9)	38 (23.8)
Statistical analysis	36 (22.5)	15 (9.4)	19 (11.9)	23 (14.4)	67 (41.9)	31 (19.4)	25 (15.6)	40 (25.0)	36 (22.5)	28 (17.5)
Drawing conclusions from research	3 (1.9)	1 (0.6)	4 (2.5)	29 (18.1)	123 (76.9)	5 (3.1)	4 (2.5)	26 (16.3)	68 (42.5)	57 (35.6)
Writing a research report	4 (2.5)	1 (0.6)	2 (1.3)	14 (8.8)	139 (86.9)	5 (3.1)	4 (2.5)	16 (10.0)	57 (35.6)	78 (48.8)
Orally presenting research findings	11 (6.9)	10 (6.3)	5 (3.1)	16 (10.0)	118 (73.8)	9 (5.6)	18 (11.3)	32 (20.0)	52 (32.5)	49 (30.6)
Overall research process^a^	5 (3.1)	2 (1.3)	20 (12.5)	38 (23.8)	95 (59.4)	0 (0.0)	3 (2.6)	23 (19.7)	49 (41.9)	42 (35.9)

^a^ Data are from 160 student dietitians, except for perceptions of the extent to which undertaking the research project contributed to the development of their research skills in the ‘overall research process’, which is from 117 students as this question was only introduced from the second cohort onwards.

Over three‐quarters of students (77.8%) reported that undertaking their research project had contributed ‘a lot’ or ‘a great deal’ to the development of their overall research skills (Table 5). The majority reported that the research project had contributed ‘a lot’ or ‘a great deal’ to their skills in: ‘completing a literature search’ (76.9%), ‘collecting new data’ (55.7%), ‘writing a research report’ (84.4%), ‘drawing conclusions from research’ (78.1%) and ‘orally presenting research findings’ (63.1%).

### Factors associated with high confidence and improved confidence

In total, 79 (49.4%) students had high confidence in their overall research skills after the research project, and the odds of this outcome was analysed using logistic regression (Table 6). Following univariable logistic regression, the only statistically significant factors were the project being an ‘external collaborative project’ (odds ratio [OR] 2.05, 95% confidence interval [CI] 1.05–4.00, *p* = 0.035) and the student reporting ‘high levels of involvement in the overall research process’ (OR = 5.51, 95% CI = 1.97–15.45, *p* = 0.001). However, following multivariable logistic regression the only factor significantly associated with high confidence in overall research skills was having ‘high levels of involvement in the overall research process’ (OR = 6.13, 95% CI = 2.03–18.49, *p* = 0.001). The multivariable regression model was statistically significant (χ^2^ = 18.244, *df* = 7, *p* = 0.011).

**Table 6:** Univariable and multivariable analysis of participant and project‐related factors associated with having ‘high confidence in overall research skills’ and having ‘improved confidence in overall research skills’ after completing the research project among 160 student dietitians

	High confidence in overall research skills after project^a^				Improved confidence in overall research skills after project^b^			
	Univariable analysis		Multivariable analysis		Univariable analysis		Multivariable analysis	
Variable (reference group)	OR (95% CI)	*p* value	OR (95% CI)	*p* value	OR (95% CI)	*p* value	OR (95% CI)	*p* value
Age	0.98 (0.93–1.02)	0.284	–	–	0.96 (0.91–1.00)	0.071	0.97 (0.92–1.02)	0.245
Gender (Female)								
Male	1.25 (0.37–4.27)	0.723	–	–	0.47 (0.14–1.63)	0.234	–	–
University (London Metropolitan)								
King's College London	1.16 (0.50–2.32)	0.668	–	–	**2.27 (1.10–4.71)**	**0.027**	1.81 (0.73–4.51)	0.202
Previous research involvement (None)								
Some previous research involvement	1.80 (0.76–4.26)	0.179	‐	‐	0.92 (0.37–2.30)	0.865	‐	‐
Supervision model/location (Internal)								
External project	**2.05 (1.05**–**4.00)**	**0.035**	2.11 (0.81–5.47)	0.126	1.49 (0.71–3.14)	0.295	0.98 (0.33–2.89)	0.969
Project type (Literature review)								
Experimental laboratory	1.25 (0.47–3.32)	0.660	2.26 (0.51–10.10)	0.285	2.54 (0.88–7.37)	0.085	1.92 (0.36–10.39)	0.448
Questionnaire or dietary survey	1.67 (0.70–3.95)	0.246	1.45 (0.44–4.71)	0.540	2.18 (0.88–5.37)	0.091	1.38 (0.38–5.01)	0.622
Clinical audit	2.56 (0.89–7.32)	0.080	1.77 (0.35–8.86)	0.488	2.43 (0.79–7.47)	0.122	1.87 (0.32–11.12)	0.489
Clinical trial or feeding study	1.69 (0.58–4.94)	0.341	2.35 (0.56–9.91)	0.244	3.44 (0.97–12.17)	0.055	2.89 (0.50–16.58)	0.234
Data collection (No data collection)								
Original data collection included	1.40 (0.74–2.67)	0.305	0.59 (0.19–1.82)	0.357	**2.05 (1.02**–**4.11)**	**0.043**	1.35 (0.39–4.72)	0.641
Involvement in overall process (Low)								
High involvement in overall process	**5.51 (1.97**–**15.45)**	**0.001**	**6.13 (2.03**–**18.49)**	**0.001**	2.24 (0.96–5.25)	0.063	2.17 (0.80–5.90)	0.128
Overall model			***χ*** **2 (7)** **=** **18.244**	**0.011**			*χ* ^2^ (9) = 14.955	0.092

Statistically significant differences are highlighted in bold.

^a^ 'High confidence' in overall research skills represents those reporting either ‘4, A lot’ or ‘5, A great deal’ of confidence in their overall research skills after completing their research project

^b^ 'Improved confidence' in overall research skills represents those whose confidence in their overall research skills increased by one or more on the Likert scale after completing their research project.

Abbreviations: CI, confidence interval; OR, odds ratio.

In total, 113 (70.6%) students reported improvement in their overall research skills after the project, and the odds of this outcome was also analysed using logistic regression (Table 6). Following univariate logistic regression, the only statistically significant factors were ‘university’ (King's College London OR = 2.27, 95% CI = 1.10–4.71, *p* = 0.027) and ‘undertaking original data collection’ (OR = 2.05, 95% CI = 1.02–4.11, *p* = 0.043). However, following multivariable logistic regression, no factors were statistically significant associated with having improved confidence in research skills and the model itself was not statistically significant (χ^2^ = 14.955, *df* = 9, *p* = 0.092).

## DISCUSSION

Research is a cornerstone of dietetic practice, and a lack of confidence in research skills is a commonly reported barrier to dietitians undertaking research.^14^, ^21^, ^25^ Research skills are commonly taught at university by experiential learning involving students undertaking a research project. The present study aimed to investigate attitudes towards research and confidence in research skills among student dietitians before and after a research project.

Students held largely positive attitudes towards research, both at baseline and after the research project (Table 2). Indeed, the only statement for which the majority did not hold positive views was ‘seeing patients is more important than research’, for which 44%–45% reported neutral (‘neither agree, nor disagree’) rather than negative views, reflecting the reality that neither seeing patients, nor doing research are more important. The attitudes towards research reported by student dietitians were, in general, similar or more positive than those reported by registered dietitians in a previous survey from which the questionnaire was taken.^14^ For example, after completing their project, there were great proportions of students agreeing that ‘Research should be carried out by all dietitians’ (students 59.4%, dietitians 38%) and disagreeing that ‘I don't see research as part of a dietitian's job’ (students 75.6%, dietitians 60%).^14^ Overwhelmingly positive attitudes to research at baseline might reflect students’ early introduction to the importance of research at university, their ongoing exposure to research through coursework and direct contact with researchers within their university, at the same time as the lack of exposure to some of the barriers to undertaking research within the practice setting.

Improvements in attitudes were only demonstrated in a small proportion of students during the research project and the number of students with a positive attitude after the project was rarely statistically significantly greater than before the project. This may be a result of ceiling effects because of the high proportion of positive responses to attitude statements observed at baseline or perhaps because their research project has given them realistic expectations of research involvement within dietetics. The only statistically significant improvement in attitudes occurred for the statement ‘I find it hard to interpret research findings’, which notably was the only attitude related to students’ own skills as opposed to addressing general attitudes towards research among dietitians.

Participation in the research project had little impact on expectations for future research involvement (Table 3). This contrasts with many studies in different countries or different disciplines. For example, evaluations of student dietitians in Australia,^3^ student dietitians and dietitians in the USA,^32^ science students in the USA,^33^ and medical and allied health students in South Africa^34^ have reported a university research project as a driver for future research involvement, including the prospect of future PhD studies. However, in these examples, undertaking a research project at university is generally elective, and therefore may select students already interested in developing a research career, whereas, in the present study, the research project module was compulsory. As well as expectations for future research involvement, a previous study across a range of disciplines has reported that university research projects increase students’ self‐reported preparedness and opportunities for a postgraduate research career,^35^ although this was not measured in the present study.

Undertaking a research project improved confidence in research skills in many students and in many aspects of the research process and was evident both when students were asked to rate their own research skills (Table 4), as well as for their perception of the extent to which the project enhanced their research skills (Table 5). More than half of students reported improvements in confidence for nine of the 10 research skills and, at the end of the project, almost half reported high levels of confidence (49.4%) and almost three‐quarters (70.6%) had improved confidence in the overall research process.

These findings concur with a previous large study in over 10,000 students in the USA from science, technology, engineering, medicine and social sciences, where 83% reported improved confidence in research skills following a university research project.^36^ Within dietetics, a relatively small health discipline, there are inevitably only much smaller studies. Positive effects on research skill development following participation in a research project has been reported in 18 student dietitians in Australia,^30^ 13 student dietitians volunteering as research assistants in the USA^37^ and 55 student dietitians undertaking an online simulated research project in the USA.^15^

The present study is therefore the largest analysis to date demonstrating the positive effects on research skills in student dietitians and, importantly, enabled an exploration of the factors associated with confidence. Many project‐related factors were explored, including the supervision model/location (internal vs. external), the type of project (e.g., experimental laboratory, dietary survey, etc.) and whether original data was collected, many of which are themselves related, highlighting the importance of multivariable regression analysis to test for independent associations. Indeed, the only factor independently associated with high confidence in the overall research process was having high levels of involvement in the overall research process. This has been previously alluded to in the wider literature outside of health professional education. For example, a previous study shows greater perceived benefit to skills when undertaking research projects of greater duration,^38^ whereas another study reports that scores on a research proposal assessment at the start of graduate school was associated with the duration and autonomy of experience during their undergraduate research project.^39^

It is encouraging that the only factor related to high confidence in overall research skills was high levels of involvement in the overall research process, which resulted in a more than six‐fold greater odds of high confidence and was more important than other project‐related factors (e.g., supervision model, type of project, collection of original data). Offering variety in opportunities in project type can be challenging in some university settings as a result of access to laboratory facilities, the research interests of academic staff and the existence of established external collaborations. However, the present study reinforces that, irrespective of these other factors, the most important goal is to offer research projects that enable as much involvement in the overall research process as possible, thus emphasising the role of experiential learning (learning by doing) in research skills development.

Reassuringly, personal characteristics (age, gender, university, previous research experience) were not significantly associated with high confidence or improved confidence. Two project‐related factors are worthy of comment: the supervision model/location and the project type. Projects that were undertaken externally and supervised by both an internal and an external supervisor were associated with greater odds of high confidence on univariable but not multivariable analysis. The positive effect of collaborative supervision is congruent with previous studies.^31^, ^40^, ^41^ The type of project undertaken was not significantly associated with high confidence or improved confidence on either univariate or multivariate analysis. Clinical audit, a common method of data collection and evaluation in dietetic practice, resulted in similar levels of high confidence and improved confidence as other project types. However, given the five different categories of project type, the study may have been underpowered to detect association with such low event rate.

Research projects vary widely in the extent to which they involve different activities in the research process. Given the relationship between high levels of involvement and high confidence in research skills, the limited confidence in specific skills such as ‘developing a hypothesis’ and ‘obtaining research ethics approval’ is unsurprising. The short duration of the research project (usually 3–4 months part time) means that students are seldom involved in the early stages of project conception, design and approval. As such, these areas where involvement and confidence are low, may benefit from being further incorporated into the teaching curriculum and into future research projects. In addition, on circulating research project titles, supervisors could indicate the likely levels of involvement in different aspects of the research process so that students can select a project more likely to fit their learning needs, as well as their interests.

Undertaking a final year research project has been described as a ‘high impact educational experience’^42^ but requires intensive and often 1:1 supervision from a university academic. Indeed, it has been argued that it is not only the activities of the research project that promote learning, but also the intensive supervision, the close interactions with academics and researchers, and the integration of students into the research fabric of university life that provides a unique educational experience unlike that of lectures and seminars.^35^ Despite this, competing curricula demands has been reported as the most common barrier to teaching research skills in pre‐registration programmes in nursing, midwifery and allied health.^43^ Given the intensive nature of research project supervision and the busy curricula in dietetic education, it is important that there are demonstrable educational outcomes to justify research projects as a highly effective method of teaching research skills – justification that this evaluation now provides.

In the present study, all students undertook a research project; however, it would be interesting to compare the attitudes and confidence in research skills of those who do (intervention) and do not (control) undertake a research project. Such a control group would be challenging in the UK environment where a research project is commonly a compulsory component in the final year of a Bachelors degree in dietetics. In other countries, including Australia, Canada and the USA, such comparisons can be made; however, students are not randomly selected to do a research project or not, and so selection bias might result in the most research‐orientated students carrying out a research project and therefore would impact conclusions.

### Strengths and limitations

This is the largest study directly comparing student dietitians’ attitudes to research and confidence in their research skills before and after undertaking a research project. It has used identical questionnaires items and response sets at baseline and after the project to enable direct comparison. However, the questionnaires were not validated prior to use, although the attitude statements were adapted from a previous survey investigating dietitians’ attitudes towards research.^14^ The lack of validity and reliability testing may introduce variation in student responses, whereas social desirability bias may lead some students to exaggerate attitudes and confidence, although statistical analysis was still able to identify findings that were statistically significant and theoretically plausible. Different questionnaires have subsequently been used to measure attitudes to research^13^, ^32^ and confidence in research skills^19^ and some instruments have undergone psychometric testing of phenomena including research involvement,^44^, ^45^ and such questionnaires may be used in future studies.

The large sample size enabled investigation of the factors independently associated with high confidence and improved confidence through multivariable logistic regression. The lack of exclusion criteria and high response rate (81.6%) minimised selection bias. Using a complete case analysis of only those students who completed a questionnaire at both time points avoided the need for data imputation, although this may have resulted in the inclusion of only those students who successfully completed the research project module, and therefore may have inflated improvements in confidence in research skills. The sample was mostly young and mostly female, although this is largely representative of student dietitians in the UK. Although the questionnaire surveys were conducted contemporaneously to the research projects, these were conducted more than 10 years ago. However, we do not consider this will impact data integrity and interpretation because the research project modules are still undertaken in the same style at the two universities and there is no reason to assume that student cohorts have changed during this period. Furthermore, this evaluation was performed at only two universities and, although there may be differences in the content and delivery of research training provided compared to other universities, the quality of the research training in pre‐registration dietetics programmes is likely representative of other universities in the UK.

The present study is also limited in that it only measured students’ self‐reported confidence in research skills. Student reports of research skills has been shown to lack agreement with academic and performance‐based assessment of research skills.^46^ One study has shown that students who had previously undertaken a research project as an undergraduate had higher scores for a research proposal assessment at the start of their PhD compared to those who had not undertaken such a project.^39^ Academic or performance‐based assessments were not used in the present study because using official university grades awarded for the research project module may have limited participation in this evaluation and because students’ perceptions of their learning remain an important educational outcome of a reflective practitioner. However, tangible outcomes, such as the number of research projects that students then presented at conferences or published as full manuscripts, or the number of students progressing to Masters or Doctoral degrees, would be ‘real‐world’ outcomes following completion of a university research project and should be included in future studies in this area.

Finally, the data collected were exclusively quantitative. Qualitative enquiries may help bridge the gap between the generally positive attitudes toward research and actual research involvement, as well as provide in‐depth insight into how the research project may impact attitudes to research and confidence in research skills.

## CONCLUSIONS

Student dietitians exhibit generally positive attitudes towards research. In general, undertaking a research project did not improve attitudes to research but did improve students’ confidence in their research skills. High levels of involvement in the overall research process was the only significant factor associated with high confidence in overall research skills. This suggests that, regardless of personal or project‐related characteristics, so long as student dietitians are highly involved in the research process, they have six‐fold greater odds of achieving high confidence after undertaking a research project. Whether these improvements in confidence are sustained and translate into future research involvement in practice warrants further investigation.

## AUTHOR CONTRIBUTIONS

*Conceptualisation, study design, and data collection*: Kevin Whelan, Jane E. Thomas and Angela M. Madden. *Data analysis and interpretation*: Kevin Whelan, Kate R. Castelli, Camilla Trizio and Oliver Howard. *Manuscript preparation*: Kevin Whelan. *Manuscript review and approval*: Kevin Whelan, Kate R. Castelli, Camilla Trizio, Oliver Howard, Jane E. Thomas and Angela M. Madden. All authors critically reviewed the manuscript and approved the final version submitted for publication.

## CONFLICTS OF INTEREST

The authors declare no conflicts of interest.

## TRANSPARENCY DECLARATION

The authors affirm that this manuscript is an honest, accurate and transparent account of the study being reported. The authors affirm that no important aspects of the study have been omitted and that any discrepancies from the study as planned have been explained.

## ETHICAL STATEMENT

The study received ethical approval from the research ethics committees of both King's College London and London Metropolitan University.
